# Genome Sequence of the Food Spoilage Yeast *Zygosaccharomyces bailii* CLIB 213^T^

**DOI:** 10.1128/genomeA.00606-13

**Published:** 2013-08-22

**Authors:** Virginie Galeote, Frédéric Bigey, Hugo Devillers, Cécile Neuvéglise, Sylvie Dequin

**Affiliations:** INRA, UMR1083 Sciences Pour l’œnologie, Montpellier, Francea; SupAgro, UMR1083 Sciences Pour l’œnologie, Montpellier, Franceb; Université Montpellier I, UMR1083 Sciences Pour l’œnologie, Montpellier, Francec; INRA, UMR 1319 Micalis, Bât. CBAI, Thiverval-Grignon, Franced; AgroParisTech, UMR 1319 Micalis, Bât. CBAI, Thiverval-Grignon, Francee

## Abstract

The ascomycetous yeast *Zygosaccharomyces bailii* is one of the most problematic spoilage yeasts in food and beverage industries, due to its exceptional resistance to various stresses. A better understanding of the molecular mechanisms underlying these stress resistance phenotypes might help develop strategies to improve food quality. Thus, we determined and annotated the genome sequence of the strain *Z. bailii* CLIB 213^T^ (= CBS 680).

## GENOME ANNOUNCEMENT

The genus *Zygosaccharomyces* belongs to the hemiascomycetous yeast phylum and includes six previously described species (*Zygosaccharomyces bailii*, *Zygosaccharomyces bisporus*, *Zygosaccharomyces kombuchaensis*, *Zygosaccharomyces lentus*, *Zygosaccharomyces mellis*, and *Zygosaccharomyces rouxii*) (1) and six recently proposed novel species (2–6). *Z. bailii* is widespread in food spoilage because of its unusual physiological characteristics, such as resistance to weak-acid preservatives and the ability to adapt to high sugar concentrations and high temperatures, vigorously ferment sugar, and grow at low pH (7–9). Great phenotypic diversity has been observed among *Z. bailii* strains (10). In wine fermentation, although this species is generally considered to have detrimental effects, potentially beneficial aspects have also been proposed (10, 11). More recently, the potential of *Z. bailii* for the production of bioethanol has also been reported (12).

While *Z. bailii* is frequently encountered in wines, it has been shown to be the donor of a 17-kb DNA region to *Saccharomyces cerevisiae*. This transferred region was first discovered in the genome of the commercial wine yeast strain *S. cerevisiae* EC1118 and thereafter was widely found in other *S. cerevisiae* wine yeast genomes, in multiple copies and showing different structures and organization (13, 14). Such eukaryote-to-eukaryote gene transfer is remarkable, given the phylogenetic distance between these two species and the large amount of DNA transferred. The *Zygosaccharomyces* genus has been poorly investigated, and so only the *Z. rouxii* genome sequence is available. Sequencing the genome of *Z. bailii* strain CLIB 213^T^ may therefore give clues to better control and exploit this species in the context of food quality improvement and biotechnological applications.

The draft genome was sequenced by using the Illumina HiSeq2000 platform (2 × 100-bp, sequencing depth, 250×), with a total of 50,868,918 reads hard-clipped to 75 bp and processed using SOAP*denovo* v1.05 (15) using a *k*-mer size of 51. A high-contiguity assembly was obtained of 212 contigs with an N_50_ contig length of 226,975 bp, further assembled into 56 scaffolds for a total size of 10,361,356 bp (N_50_ scaffold length of 932,251 bp). Twenty-seven scaffolds of >10 kb (cumulative size of 10,268,813 bp with a 42.5% G+C content) were suitable for automatic annotation. A total of 5,084 putative protein-coding genes, including 217 pseudogenes, were predicted using Amadea BioPack (ISoft, France) and were further curated manually. BLASTp comparison revealed that 98.2% of these genes have a homolog in *Z. rouxii*, only 8 are species-specific genes, and 5 were acquired by interkingdom lateral transfer. A total of 162 tRNAs were identified using tRNAscan-SE v1.3.1 (16).

The 17-kb gene cluster transferred to *S. cerevisiae* was found in a single copy in *Z. bailii*, suggesting that the amplification is specific to *S. cerevisiae*. Genes encoding key enzymes for glucose and fructose assimilation were identified. The *FFZ1* gene, encoding a low-affinity facilitator protein specific for fructose, previously identified in *Z. bailii* strain ISA1307 (17), was found on scaffold 5. We also identified a gene encoding a protein that is highly similar (77% identity) to EC1118 Fsy1p, a high-affinity fructose/H^+^ symporter (18). Three other genes showed a high similarity with *S. cerevisiae* Hxtp glucose transporters. The large number of genes that potentially encode sugar transporters with different characteristics might explain the high fermentative performance of *Z. bailii*.

### Nucleotide sequence accession numbers. 

The sequence of the *Z. bailii* genome has been deposited at EMBL under the accession no. HG316454 to HG316480 (as 27 scaffolds).
